# Vaginal *Lactobacillus gasseri* biosurfactant: a novel bio- and eco-compatible anti-*Candida* agent

**DOI:** 10.1016/j.bioflm.2025.100299

**Published:** 2025-06-24

**Authors:** Federica Monti, Barbara Giordani, Stefano Fedi, Daniele Ghezzi, Paola Galletti, Laura Mercolini, Roberto Mandrioli, Carola Parolin, Barbara Luppi, Beatrice Vitali

**Affiliations:** a Department of Pharmacy and Biotechnology, University of Bologna, Bologna, 40127, Italy; b Department of Chemistry Giacomo Ciamician, University of Bologna, Bologna, 40126, Italy; c Department for Life Quality Studies, University of Bologna, Rimini, 47921, Italy

**Keywords:** Biosurfactant, Vaginal microbiota, Lactobacilli, Candida, Biofilm

## Abstract

Vulvovaginal candidiasis (VVC) and recurrent vulvovaginal candidiasis (RVVC), caused by *Candida* spp. overgrowth, are common and challenging infections of the genital tract. Among *Candida* virulence factors, the ability to adhere to host epithelium and to form biofilms are frequently retrieved, especially in RVVC. Vaginal pathogen overgrowth is counteracted by resident lactobacilli, which exert a barrier thanks to the production of antimicrobial metabolites, such as biosurfactants (BS).

BS was recovered from vaginal *Lactobacillus gasseri* BC12 and its chemical characteristics as well as its ability to lower the surface tension and to emulsify two different immiscible phases were investigated. BS showed the typical features of a lipopeptide with a critical micellar concentration of 1.2 mg/mL. BS showed antibiofilm activity towards various *Candida albicans* and non-*albicans* isolates, notably, it was able to prevent biofilm formation and eradicate preformed biofilms. The absence of cytotoxicity of BS and its ability to counteract the adhesion of *Candida* spp. were highlighted on HeLa cells through MTT and competition/exclusion assays, respectively. The environmental impact of BS was also investigated on a microcosm model (spring water) by culture-based and molecular (16S rRNA-targeted Illumina sequencing) methods, and no remarkable modifications in the taxonomy composition of the bacterial ecosystem were observed.

To conclude, BS from *L. gasseri* BC12 appears as a promising, biocompatible and environmentally friendly approach to prevent and treat VVC/RVVC.

## Highlights

• Vaginal *Lactobacillus gasseri* BC12 produces a lipopeptidic biosurfactant (BS-BC12).
• BS-BC12 inhibits *Candida albicans* and non-*albicans* biofilms.
• BS-BC12 impairs *Candida albicans* and non-*albicans* adhesion to HeLa cells.
• BS-BC12 is not cytotoxic, and it is eco-compatible.
• BS-BC12 does not impact culturable bacterial counts in microcosms.

## 1 Introduction

Vulvovaginal candidiasis (VVC) is one of the most common infections of the lower genital tract. This infection usually manifests with characteristic symptoms like pain and soreness of the vagina and vulva, burning and copious secretions, and discomfort during urination and sexual intercourse [1]. It is estimated that 75 % of women, during their life, experience VVC at least once and this occurs more frequently between the ages of 25 and 34. The primary causative agent is *Candida albicans*, an opportunistic pathogen reported in most cases of VVC in the last decades [2,3]. However, the occurrence of non-*albicans Candida* (NAC) infections, caused by species such as *C. glabrata*, *C. krusei*, and *C. parapsilosis,* has recently increased. The reason for this trend could be a higher frequency of drug resistance cases detectable in different species of the *Candida* genus. The excessive or wrong use of antimycotic drugs is one of the possible causes of reduced susceptibility or resistance of this fungus to pharmacological treatments [4].

Additionally, many *Candida* strains naturally own numerous virulence factors. These factors allow them to adhere to host epithelium strictly, evade the host immune system, and resist antifungal drugs. The ability of microorganisms to form biofilms, three-dimensional and multi-layered structures where microbial cells live in association with each other immersed in an extracellular matrix, is influenced by many intrinsic factors, including the expression of genes that encode for adhesion proteins [5]. In particular, the ability to adhere to the host cells and to form biofilms has been reported in several *Candida* isolates [2]. Biofilm structure works as a protective shield against host immune system and drugs, and this makes *Candida* infections extremely difficult to get rid of and could be related to recurrent infections [5,6]. Recurrent vulvovaginal candidiasis (RVVC, defines as three or more symptomatic episodes in 12 months) is a recalcitrant and difficult-to-cure fungal infection that affects quality of life of many women [7]. While the *in vivo* and *in situ* presence of *Candida* biofilm in VVC is highly debated [8,9], *Candida* biofilms have been found on the vaginal epithelium of patients with RVVC, demonstrating the important role of biofilm formation in the persistence of the disease [10].

*Lactobacillus* is the most abundant genus of vaginal microbiota of women of childbearing age, and the most represented vaginal species are *Lactobacillus crispatus*, *Lactobacillus gasseri*, *Lactobacillus iners* and *Lactobacillus jensenii* [11]. These microorganisms are commonly linked with women’s health and play several essential functions for their host: they can exclude competitive pathogens, adhere and remain attached to the epithelium, regulate the host immune response and produce antimicrobial metabolites [[12], [13], [14], [15]]. Among these metabolites, biosurfactants (BS) are noteworthy: they are amphipathic molecules with different chemical structures, commonly produced by microorganisms as secondary metabolites. BS play many advantageous functions for the producers since they promote the transport of nutrients, take part in interactions with the host, other microorganisms and the external environment and act as antimicrobial factors [16]. In this regard, previous studies have shown that BS from vaginal *Lactobacillus* strains exert anti-biofilm activity against some pathogens [17,18] and can interfere with the epithelial adhesion of *Candida* [19,20], resulting in prospective, attractive biotherapeutic agents to address the challenge of antibiotic resistance. In recent years, numerous studies have focused on bacterial-derived BS for many interesting features, such as their low toxicity and high specificity, and above all their bio- and eco-compatibility. All these characteristics, coupled with their “green” source, make BS a non-harmful and eco-friendly substitute for chemically synthesised surfactants [21,22].

The present study describes the production of BS by *L. gasseri* BC12 (BS-BC12), a strain previously isolated from the vagina of a healthy premenopausal woman [23]. BS-BC12 was characterized in terms of chemical structure and technological properties, such as critical micellar concentration and emulsification index. Subsequently, we focused on the ability of BS-BC12 to exert anti-biofilm and anti-adhesive activities towards *Candida albicans* and non-*albicans* species. Furthermore, BS-BC12 was tested on HeLa cells to evaluate the biocompatibility of this molecule with cervical epithelium. We also focused on BS impact on the micro-environment, particularly on a fluvial micro-habitat, taken as a model, to evaluate its eco-compatibility. Specifically, microcosms with spring water were set up and cultivable bacteria were counted at different pre-determined time points. In parallel, a phylogenetic characterization with Next Generation Sequencing (NGS) technology was carried out. Thus, it was possible to supervise qualitative and quantitative changes within the bacterial community over time due to the presence of BS.

## 2 Materials and methods

### 2.1 Microorganisms and culture conditions

*L. gasseri* BC12, previously isolated from the vaginal swab of a healthy premenopausal woman [23], was cultured in de Man, Rogosa and Sharpe (MRS) broth (Becton, Dickinson and Co., Sparks, USA) supplemented with 0.05 % l-cysteine in anaerobic jars containing GasPak EZ for 24 h at 37 °C.

*Candida* strains used in the present study belong to the species *C. albicans* (SO1, SO6, SO8, SO10, DSM1386), *C. glabrata* (SO17, SO18), *C. lusitaniae* (SO22), *C. krusei* (SO26) and *C. parapsilosis* (SO27). *C. albicans* DSM1386 was purchased from DSMZ (Braunschweig, Germany). The other *Candida* strains belong to a collection of yeasts isolated from vaginal swabs of VVC affected-premenopausal women during routine diagnostic procedures at the Microbiology Laboratory of Sant’Orsola-Malpighi University Hospital of Bologna, Italy [23]. *Candida* strains were cultured aerobically in Sabourad Dextrose (SD) agar plates (Becton, Dickinson and Co., Sparks, USA) for 24 h at 32 °C.

### 2.2 Isolation of BS from *L. gasseri* BC12

The cell-bound BS was isolated following the protocol previously described [19], with few modifications. Briefly, 90 mL of an overnight culture of *L. gasseri* BC12 were inoculated in 900 mL of MRS broth and cultured for 24 h at 37 °C. The microbial suspension (ca 10^9^ CFU/mL) was centrifuged at 5000×*g* for 15 min (Centrisart® D-16C Sartorius, Göttingen, Germany), the cell pellet (10^12^ CFU) was washed twice, resuspended in 300 mL of sterile PBS (2.38 g/L Na_2_HPO_4_, 0.19 g/L KH_2_PO_4_ and 8 g/L NaCl) and stirred at 130 rpm for 2 h at room temperature to allow the release of cell-bound BS.

Afterwards, the sample was centrifuged (8000×*g* for 30 min), and the supernatant was filtered through a 0.22 μm pore size filter, then subjected to dialysis against demineralized water in Cellu-Sep© membrane (molecular weight cut-off 6000–8000 Da) for 24 h at room temperature and freeze-dried at 0.4 atm and −56 °C.

### 2.3 Chemical characterization

To identify different types of chemical bonds and functional groups of the freeze-dried BS-BC12 Fourier transform infrared spectroscopy (Jasco FT-IR 4100 spectrophotometer) was used. The BS was triturated with KBr powder in a weight ratio of 1:10 and pressed with a hydraulic press at a pressure of 70 tons for 5 min. The obtained discs were scanned between 4000 and 450 cm^−1^.

Tandem mass spectrometry (MS/MS) assays were carried out according to the previously published method by Abruzzo et al. [19]. Briefly, a Waters (Milford, MA, USA) Micromass Quattro Micro triple quadrupole (QqQ) mass spectrometer was used and interfaced with an electrospray ion source operating in positive and negative ionization modes (ESI+/ESI−). BS was dissolved up to a concentration of 1 μg/mL in different solvents (ultrapure water, methanol, acetonitrile, their mixtures at different ratios, and their acidified or basified mixtures). MS and MS/MS spectra (scan duration: 500 m s, *m/z* range: 20–3000 Da/e) were acquired by applying MS parameters as follows: the capillary voltage was set at 3.0 kV, while cone voltage was tested within a range of 15–100 V; source and desolvation temperatures were kept at 120 °C and 150 °C, respectively; cone gas (N_2_) flow was set to 50 L/h while desolvation gas (N_2_) flow was 200 L/h.

UHPLC-HRMS analyses were carried out on a Waters Xevo system which included a Xevo G2 Q-TOF HRMS detector. Separation was achieved on an Acquity – BEH C (C18, 50 × 2.1 mm I.D., 1.7 μm) column using a mobile phase composed of 0.3 % formic acid in water (solvent A) and 0.3 % formic acid in acetonitrile (solvent B). The flow rate was 0.2 mL/min, and a 5–80 % linear gradient of solvent B in 35 min was applied. The injection volume was 5 μL. ESI + operating parameters were the same as for MS scans, except capillary voltage 3.0 kV, cone voltage 55 V. Spectral scans were carried out in the 100-16,000 *m/z* range.

The flash combustion technique determined CHNS content of BS-BC12 (lyophilized sample) using an elemental analyzer (Thermo Scientific, Flash 2000, organic elemental analyzer). Samples were analyzed in duplicate using BBOT for the calibration curve.

### 2.4 Surface-activity determination and critical micelle concentration

The critical micellar concentration (CMC) of BS-BC12 was measured using the Ring method, as previously described [19]. Briefly, surface tension (dyne/cm) of different concentrations of dialysed and freeze-dried BS (0.3–4.0 mg/mL) was measured at room temperature using a tensiometer (K8600E Krüss GmbH, Hamburg, Germany) equipped with a 1.9 cm platinum ring.

### 2.5 Emulsification property of BS-BC12

To determine the emulsification index (EI_24_), a mixture of 1 mL of BS aqueous solution (1 mg/mL), 4 mL of distilled water and 6 mL of oil (olive oil, sunflower oil or wheat germ oil) was vigorously shaken in graduated glass test tubes for 2 min in order to obtain maximum emulsification [24]. The height of the emulsion (EI_24_) was measured after 24 h. Distilled water and Tween 80 (1 mg/mL) were used as negative and positive controls, respectively.

### 2.6 Anti-biofilm activity of BS-BC12

The anti-biofilm activity of BS-BC12 solubilized in sterile water toward *Candida* strains was evaluated by considering two different mechanisms of action: the inhibition of biofilm formation and the eradication of pre-formed biofilm. The anti-biofilm activities were evaluated as previously described [18], with few modifications. BS-BC12 was solubilized in sterile distilled water at a final concentration of 1.2 mg/mL. Overnight grown *Candida* strains biomass was resuspended in SD broth to a final concentration of 10^7^ CFU/mL.

For the inhibition assay, sterile 96 multi-well flat-bottomed plates (Falcon®, Corning, USA) were filled with 100 μL of *Candida* suspension and 100 μL of BS-BC12 solution. Positive controls contained only 100 μL of yeast suspension and 100 μL of sterile distilled water; blank wells were filled with 100 μL of water and 100 μL of SD broth. The plates were incubated at 32 °C for 72 h, wells were emptied, and biofilm formation was evaluated through crystal violet staining. Adherent cells were washed once with saline (0.9 % NaCl *w/v*), fixed with 100 μL of methanol for 10 min and stained with 1 % crystal violet (*w/v*) in 12 % ethanol for 10 min. Afterwards, wells were washed three times with saline to remove the excess stain. To quantify biofilm formation, 200 μL of ethanol were added to each well, and the OD_595_ read was carried out with an EnSpire Multimode Plate Reader (PerkinElmer Inc., Waltham, MA).

For the eradication assay, 96 multi-well plates were inoculated with 200 μL of *Candida* suspensions and incubated at 32 °C for 72 h; blank wells were filled only with 200 μL of SD. Afterwards, wells were emptied and washed once with sterile saline to leave only adherent cells. Then, biofilms were treated with 100 μL of SD medium and 100 μL of BS-BC12. Positive controls and blanks were filled with 100 μL of SD and 100 μL of sterile distilled water. Plates were incubated for additional 48 h at 32 °C, and then the biofilm quantification was performed following the protocol described above.

### 2.7 Effects of BS-BC12 against *Candida* planktonic cultures

BS-BC12 solubilized in distilled water (100 μL) was incubated along with 100 μL of *Candida* suspension (1 × 10^5^ CFU/mL) in sterile 96 multi-well flat-bottomed plates. Two concentrations of BS-BC12 were tested (1.2 and 0.6 mg/mL). Wells filled with 100 μL of *Candida* suspension and 100 μL of sterile distilled water served as positive control. Wells were filled with 100 μL of water and 100 μL of SD broth served as blanks and sterility control. Plates were incubated at 32 °C and the growth was evaluated after 24 h by reading the turbidity (OD_600_) with an EnSpire Multimode Plate Reader.

### 2.8 Cytotoxicity of BS-BC12 on HeLa cells

The impact of BS-BC12 on the proliferation of human cervix epithelial cells (HeLa) was evaluated as previously described [20]. HeLa cells were maintained in Dulbecco’s Minimal Essential Medium (DMEM, Merck, Milan, Italy), supplemented with 1 % l-glutamine and 10 % bovine serum at 37 °C in a 5 % CO_2_ atmosphere. Cells were seeded in 96-well flat-bottomed plates (5 × 10^4^ cells/well) and incubated for 24 h. Afterwards, the exhausted medium was replaced with BS-BC12 solubilized in the appropriate cell medium at different concentrations (0.15–1.2 mg/mL), and plates were incubated for 24 h or 48 h. Cells cultured in their medium without biosurfactant were used as positive control. At the end of the incubation times, the medium was replaced with 100 μL of 3-(45-dimethylthiazol-2-yl)-2.5-diphenyltetrazol (MTT) (Merck) diluted in cell medium at the concentration of 0.5 mg/mL. Plates were incubated for 4 h at 37 °C with 5 % CO_2_. Then, the MTT solution was discarded, and 100 μL of isopropyl alcohol was added to each well to dissolve the formazan salt crystals formed [25]. After 30 min, the optical density (OD) at 570 nm was measured using an EnSpire Multimode Plate Reader (PerkinElmer Inc., Waltham, MA). Cells viability (%) in the presence of BS-BC12 was determined by comparing the OD_570_ with that of control.

### 2.9 Anti-adhesive activity of BS-BC12 toward *Candida* spp. in HeLa cells

The anti-adhesive activity of BS from *L. gasseri* BC12 toward *Candida* spp. on HeLa cells was determined as previously described [20], with few modifications. HeLa cells were grown on sterile glass coverslips placed in 24-well culture plates, up to 75 % confluence. Two different anti-adhesive mechanisms were evaluated, namely competition and exclusion. *Candida* spp. suspensions were prepared in sterile saline solution at a final concentration of 1 × 10^9^ CFU/mL. For the competition test, *Candida* suspensions (ratio 1:100 with HeLa cells) and BS-BC12 at 0.6 mg/mL concentration were contemporarily added to each well and plates were incubated for 1 h at 37 °C. For the exclusion test, HeLa cells were first incubated with BS-BC12 for 1 h at 37 °C, and then *Candida* suspensions were added for 1 h. HeLa cells incubated with *Candida* and without biosurfactant were used as control to evaluate basal fungal adhesion (100 %).

Once the incubation was finished, cells were washed with sterile PBS and stained using the May-Grünwald-Giemsa (Merck) protocol [23]. Results were determined by counting the number of fungal cells bound to HeLa using optical microscopy (1000x). Adherent *Candida* spp. counts were evaluated in at least 20 randomly chosen optical fields.

### 2.10 Experimental microcosm setup and sampling strategy

For the experiment, spring water was collected from Rio Canale in Castel D'Aiano (Bologna, Italy) (44° 17′ 41.14″ N, 10° 58′ 56.24″ E) during the summer of 2023, and stored at 4 °C for maximum one day before use. The experiments were performed in glass bottles of 500 mL capacity and filled with 200 mL of spring water. Two bottles were used as controls, while the other two bottles were added with BS-BC12 at the concentration of 50 ppm. Microcosms were sealed at room temperature and gently stirred at 100 rpm for 90 days.

On fixed days (0, 15, 30, 90 from the beginning of the experiment), 1 mL of water of each microcosm was used to count the cultivable bacteria. Enumeration was estimated by spreading 100 μL of tenfold dilutions of microcosm samples on R2A agar (AG Scientific, CA, USA) and Tripticase Soy Agar (TSA) (Becton, Dickinson and Co., Sparks, USA) plates added with cycloheximide (10 % *w/v*) to avoid fungi growth. Plates were incubated at room temperature for 72 h, and results were expressed as colony forming units (CFU) mL^−1^.

At the same time points, 20 mL of water from each microcosm were filtered on a 0.22 μm membrane under vacuum for DNA extraction, and filters were stored at −20 °C until analysis. On the day of extraction, half of each filter was cut into small pieces and homogenized using the beads provided by the DNeasy PowerSoil Kit (Qiagen, Hilden, Germany) and total DNA was extracted according to the manufacturer’s protocol with some modifications as described by Cappelletti et al. [26]. The extracted DNA was used as a template for PCR amplification targeting the V3–V4 hypervariable regions of the 16S rRNA gene using the primer pair 341F (5′-CCTACGGGAGGCAGCAG-3′) and 806R (5′-GGACTACHVGGGTWTCTAAT-3′) [27]. Each primer was modified with an Illumina adaptor sequence at the 5′ end. The amplifications were performed in 50 μL final volume containing primers 500 nM, 1x Takara Ex Taq buffer with MgCl_2_, dNTPs mix 200 μM, Takara Ex Taq Polymerase 0.5 U. The amplification program included a first DNA denaturation at 98 °C for 10 s, followed by 30 cycles of denaturation at 98 °C for 30 s, primer annealing at 55 °C for 30 s, extension at 72 °C for 30 s, and a final extension at 72 °C for 5 min. Some PCR products were then purified from electrophoresis gel using the QIAEX II® Gel Extraction Kit (Qiagen, Hilden, Germany). Samples were stored at −20 °C immediately after amplification and purification process. Amplicons were submitted to the library preparation and Illumina MiSeq sequencing platform for indexing and paired-end sequencing (2 × 250 bp; reagent kit, v2) at the sequencing service Macrogen (Macrogen Europe, The Netherlands). The output reads were first trimmed for their adapters and primer sequences and then checked for chimera and quality by using QIIME2 software version 2022.2.0. Reads were processed into Amplicon Sequence Variants (ASVs) using the DADA2 package version 1.14 as described by Ghezzi et al. [28]. Community diversity indexes calculation was performed with Primer-E version 7 (Primer-E Ltd, Plymouth, UK). The taxonomic assignment of the resulting ASVs was performed by querying the ASVs against SILVA SSU database version r138.1 [29].

### 2.11 Data analysis

Each experiment was repeated at least three times (n ≥ 3), and results were expressed as mean value ± standard deviation (SD). Student’s t-test was used to compare two means, one-way ANOVA was used for multiple comparison. Statistical analyses of alpha diversity indexes and of bacterial composition at genus level were performed on MicrobiomeAnalyst. GraphPad Prims version 10.0.2 for Windows (GraphPad Software, San Diego, CA, USA, www.graphpad.com) was employed for statistical analysis. Differences were considered significant for *P* < 0.05.

## 3 Results

### 3.1 Chemical characterization of BS-BC12

The FT-IR spectrum of the freeze-dried sample of BS isolated from *L. gasseri* BC12 is reported in Fig. S1. The pattern showed the presence of a peptide moiety combined with aliphatic groups, a characteristic feature of lipopeptide molecules. The strong peak around 3300–3400 cm^−1^ can be attributed to vibrational N–H stretching transitions, while bands at 1654 cm^−1^ and 1749 cm^−1^ can be due to C=O stretching transitions. Moreover, the absorption peak at 1541 cm^−1^ can be ascribed to the deformation mode of the N–H bond combined with the C–N stretching mode. On the other hand, the presence of aliphatic chains can be deducted by the peaks at 2958 cm^−1^ and 2928 cm^−1^ relative to CH_2_ and CH_3_ stretching, respectively. A similar FT-IR absorption spectrum is related to surfactin, a lipopeptide biosurfactant already described in literature [30].

MS full scan and MS/MS spectra were obtained in a *m/z* range between 20 and 3000 by direct infusion of BS solutions at the concentration of 1 μg/mL in H_2_O, H_2_O/MeOH, H_2_O/ACN and H_2_O/MeOH/HCOOH mixtures. These solvents were chosen based on BS solubility: the BS was completely soluble at the chosen concentration.

No significant MS spectrum was obtained in negative ionization mode under the tested conditions. This suggests a lack (or scarcity) of the acidic functions typical of glycolipids. On the other hand, full scan spectra acquired in ESI + mode showed significant signals, whose intensity increased as the cone voltage increased.

MS/MS and HRMS spectra obtained from both direct spectral scans and chromatographic peaks in the 100–16000 *m/z* range included the presence of fragments spaced by multiples of 14 *m*/*z*, typical of homologous fatty acid portions from lipid-like compounds; some signals were compatible with 16:2, 20:0, 22:1, 24:1 and 26:1 fatty acids. Low-mass signals are also present in the 50–200 *m/z* range, tentatively attributable to amino acid portions of tyrosine, proline, glycine and arginine.

The presence of BS-BC12 residual free phosphate salts prevented the determination of absolute carbon and nitrogen content. The obtained C/N ratio (elemental analysis) of 3.35 is consistent with the lipopeptide structure in which the peptidic part prevails.

### 3.2 Surface-activity determination and critical micellar concentration of BS-BC12

**Fig. 1:**
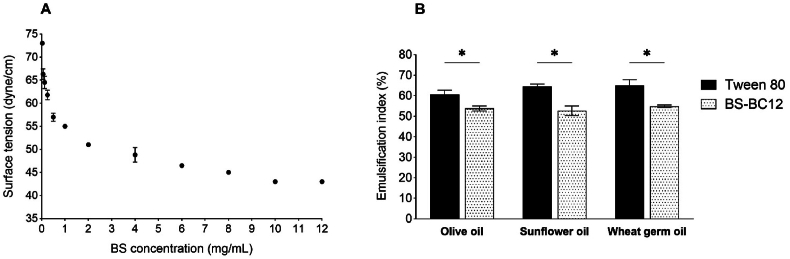
(A) Surface tension (dyne/cm) versus BS-BC12 concentration (mg/mL). **(B)** Emulsification index at 24 h (EI_24_) of Tween 80 and BS-BC12 calculated on olive oil, sunflower oil and wheat germ oil. Data are plotted as mean ± standard deviation (n = 3). Significance between Tween 80 and BS-BC12 is reported (∗*P* < 0.05).

One of the essential properties of BS is its ability to lower the surface tension of liquids. The biosurfactant produced by *L. gasseri* BC12 reduced the surface tension from 66.3 ± 1.2 to 43.0 ± 0.0 dyne/cm. Surface tension gradually decreased with the increase of BS concentration (Fig. 1A). The calculated CMC value was 1.2 mg/mL.

### 3.3 Emulsification properties of BS-BC12

The criterion to define an excellent emulsion-stabilizing capacity of a surfactant is its ability to maintain at least 50 % of the original emulsion volume 24 h after its formation [31]. The emulsification properties of BS-BC12 were tested against olive oil, sunflower oil and wheat germ oil. In all cases, EI_24_ of BS was found lower than that of Tween 80 (*P* < 0.01), used as a reference control, but higher than 50 % (Fig. 1B). Considering that the E_24_ values obtained were comparable with those reported in previous papers for other lipopeptide biosurfactants [19,32], BS from *L. gasseri* BC12 can be considered capable to exert suitable emulsification activity against different substrates.

### 3.4 Anti-biofilm activity of BS-BC12

Since the ability to form biofilm is closely associated with *Candida* pathological features [6], the anti-biofilm activity of *L. gasseri* BC12 biosurfactant was tested towards five *C. albicans* strains and five *C.* non-*albicans* strains. For the inhibition and eradication assays, 96 multi-well plates were used as abiotic surface since this yeast’s *in vitro* biofilm formation has been correlated with *in vivo* and *ex vivo* models [33]. The anti-biofilm activity was evaluated for two BS-BC12 concentrations corresponding to the critical micellar concentration (1.2 mg/mL) and its half (0.6 mg/mL).

**Fig. 2:**
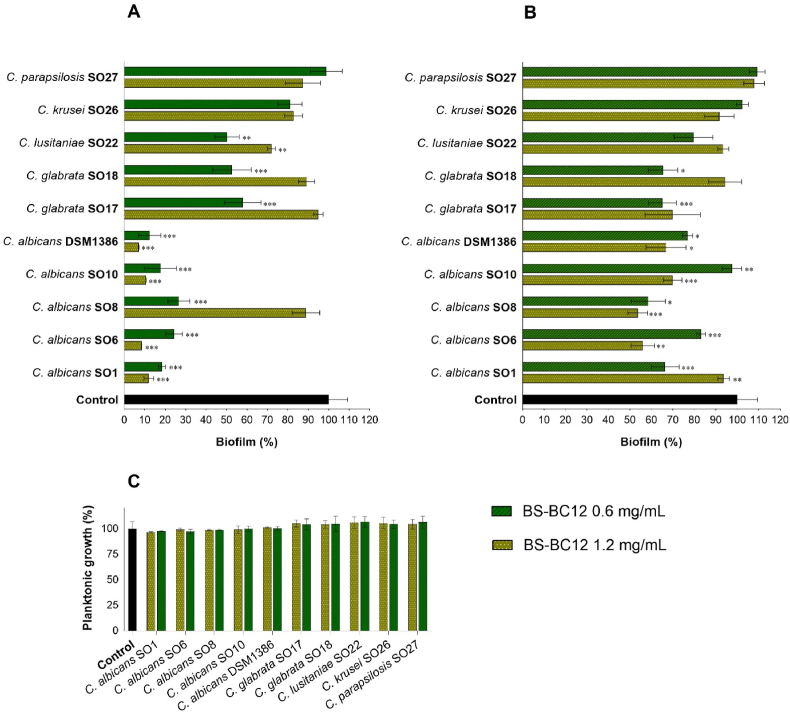
(A) Inhibition of *Candida* spp. biofilm development. Data represent biofilm formation (%) in the presence of BS-BC12 at 0.6 and 1.2 mg/mL. **(B)** Dispersal of *Candida* spp. pre-formed biofilm. Data represent residual biofilm (%) after treatment with BS-BC12 at 0.6 and 1.2 mg/mL. **(C)** Effects of BS-BC12 on planktonic cultures of *Candida* spp. Data represent the growth (%) after treatment with BS-BC12 at 0.6 and 1.2 mg/mL. For all experiments untreated control is assumed to be 100 %. Data are expressed as mean value ± standard deviation (%) (n = 3). The statistical significance with respect to untreated control was reported (*∗∗∗P* < 0.001; *∗∗P* < 0.01; *∗P* < 0.05).

For the inhibition experiments, *Candida* suspensions were incubated with BS-BC12, and biofilms were left to develop for 72 h; biofilm formation was calculated with respect to untreated wells (control) (Fig. 2A). BS-BC12, at both concentrations tested, revealed a noteworthy ability to reduce the biofilm formation of 4 *C. albicans* out of 5 strains with biofilm formation percentages always lower than 30 %. Indeed, biofilm formation values ranged from 7 % to 12 % in the presence of BS-BC12 at 1.2 mg/mL and from 12 % to 27 % with BS at 0.6 mg/mL (*P* < 0.001 vs control). BS from *L. gasseri* BC12 was less effective in inhibiting biofilm formation of non-*albicans* species, with variable results depending on *Candida* strain. No significant activity was observed on *C. krusei* SO26 and *C. parapsilosis* SO27, while the biofilm formation of *C. glabrata* SO17–SO18 and *C. lusitaniae* SO22 was 50–58 % in the presence of BS 0.6 mg/mL. The two BS-BC12 concentrations displayed a different activity spectrum: the concentration 1.2 mg/mL was more effective on *C. albicans*, while 0.6 mg/mL was more active on *C.* non-*albicans* (i.e. *C. glabrata* and *C. lusitaniae*). These results suggest that the inhibitory activity of BS from *L. gasseri* BC12 was dose- and species-dependent.

For the eradication assay, *Candida* biofilms were formed for 72 h and then treated for 48 h with BS-BC12 at the final concentrations of 1.2 mg/mL and 0.6 mg/mL. Results, reported in Fig. 2B, are expressed as biofilm growth percentages compared to untreated control. BS-BC12 at the concentration of 1.2 mg/mL resulted in effective biofilm eradication of all *C. albicans* strains except for SO1, with 30–46 % eradication rates. BS-BC12 at 0.6 mg/mL dispersed biofilms of *C. albicans* strains except for *C. albicans* SO10, with 17–42 % eradication rates. The impact of BS-BC12 on non-*albicans* species seemed to be less marked: BS-BC12 at 1.2 mg/mL was significantly effective only on *C. glabrata* SO17, reducing biofilm of 30 %, while at 0.6 mg/mL was effective on both *C. glabrata* strains and *C. lusitaniae* SO22. On these species, BS-BC12 determined a biofilm eradication ranging from 20 % to 35 %, confirming that the concentration of 0.6 mg/mL was the most effective in inhibiting the non-*albicans* species. No significant activity was observed on *C. krusei* SO26 and *C. parapsilosis* SO27 (*P* > 0.05).

### 3.5 Impact of BS-BC12 on *Candida* planktonic cultures

*L. gasseri* BC12 biosurfactant, at the same concentrations used for anti-biofilm testing (1.2 and 0.6 mg/mL), was also sought for the effects on free-floating *Candida* cells. The results are presented in Fig. 2C. No significant differences were observed compared to non-treated *Candida* (control), indicating that BS-BC12 did not exhibit direct fungistatic or fungicidal activity against *Candida* isolates.

### 3.6 Cytotoxicity of BS-BC12 on HeLa cells

**Fig. 3:**
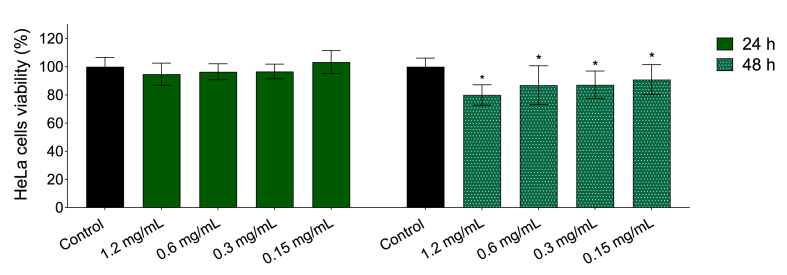
Viability of HeLa cells after 24 h and 48 h of incubation in the presence of BS-BC12 at four concentrations (0.15–1.2 mg/mL). Data are plotted as mean values ± standard deviation (n = 3). The statistical significance with respect to untreated control was reported (∗*P* < 0.05).

The cytotoxicity of the BS-BC12 was evaluated on HeLa cells, cellular model of cervico-vaginal epithelium. HeLa cells were treated with BS-BC12 at four different concentrations for 24 h and 48 h and then subjected to MTT assay (Fig. 3). At 24 h, none of the tested concentrations significantly reduced cell viability (*P* > 0.05). After 48 h, HeLa viability was slightly reduced in the presence of BS-BC12 at all tested concentrations, causing a 9–20 % decrease.

### 3.7 Anti-adhesive activity of BS-BC12 toward *Candida* spp. on HeLa cells

**Fig. 4:**
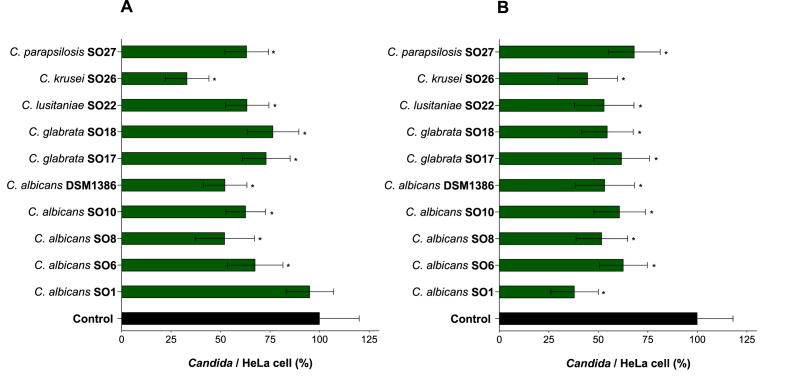
Adhesion of *Candida* spp. on HeLa cells in the presence of BS-BC12 0.6 mg/mL, evaluated in **(A)** competition and **(B)** exclusion assays. The statistical significance with respect to untreated control was reported (*∗P* < 0.05).

To evaluate the capability of BS from *L. gasseri* BC12 to prevent and inhibit the adhesion of *Candida albicans* and non-*albicans* strains to cervico-vaginal epithelium, competition and exclusion assays on HeLa cells were performed. These experiments were performed using BS-BC12 at 0.6 mg/mL concentration. Results, reported in Fig. 4, are expressed in percentages as adherent *Candida* to HeLa cells treated with BS compared with the untreated control.

In competition assay (Fig. 4A), BS from *L. gasseri* BC12 revealed a noteworthy ability to reduce the adhesion to HeLa cells of 9 *Candida* strains out of 10 strains tested. Adhesion rates of *C. albicans* (SO6, SO8, SO10 and DSM1386) was reduced by 32.5–47.9 %, whilst BS did not significantly affect adhesion of *C. albicans* SO1. Importantly, also non-*albicans* strains adhesion was susceptible to the action of BS-BC12. In particular, the highest activity was observed against *C. krusei* SO26, with a 67 % decrease of adhesion, while the adhesion percentages of the other non-*albicans* strains ranged from 63 % to 76 %.

In the exclusion assay (Fig. 4B), the cells pre-treatment with *L. gasseri* BC12 biosurfactant determined a significant adhesion decrease of all tested *Candida*. Contrary to what was previously observed for the competition assay, in the exclusion assay *C. albicans* SO1 was the most susceptible strain, since its adhesion was reduced by 62 % in the presence of BS-BC12. For the other *C. albicans* strains, adhesion rates ranged from 52 % to 63 %. Among *Candida* non-*albicans* strains, *C. krusei* SO26 exhibited the most significant decrease (55.3 %) in adhesion after the preventive treatment of HeLa cells with BS-BC12, in accordance with competition assay results. Instead, the adhesion of the other non-*albicans* species was reduced by 32–55 %. These results underline that BS from *L. gasseri* BC12 was more efficient in exclusion assay.

### 3.8 Evaluation of BS-BC12 eco-compatibility

To evaluate BS-BC12 eco-compatibility and green potential, we determined the impact of BS-BC12 on a model micro-environment represented by a fluvial micro-habitat. Specifically, microcosms with spring water were set up, and BS-BC12 was tested at the concentration of 50 ppm; then cultivable bacteria were counted at different time points. Bacterial counts were carried out on R2A and TSA plates added with cycloheximide to avoid fungi growth. TSA is a generic bacterial medium, while R2A is an enriched medium particularly suitable for environmental bacteria growth; results are reported in Table 1. Control fluvial microcosm showed, on TSA, an initial bacterial load of 1.7 × 10^4^ CFU/mL, reaching a concentration of 4.8 × 10^5^ CFU/mL after 90 days. On R2A the bacterial count was 2.6 × 10^4^ CFU/mL at T0 and 1.1 × 10^6^ at T3. Bacterial counts were slightly higher on R2A probably because this medium is more appropriate for the growth of environmental microorganism. No significant difference was seen in bacterial heterotrophic counts in microcosm added with BS-BC12 50 ppm with respect to control at any time point, suggesting that BS from *L. gasseri* BC12 did not affect the total number of cultivable bacterial population.

**Table 1:** Counts of heterotrophic cultivable bacteria grown on Tripticase Soy Agar (TSA) and R2A plates + cycloheximide in fluvial microcosms (control) added with BS-BC12 50 ppm (+BS-BC12), expressed as Colony Forming Units CFU/mL ± SD.

	TSA		R2A	
	Control	+ BS-BC12	Control	+ BS-BC12
**T0 (0 days)**	1.7 × 10^4^ ± 0.5 CFU/mL		2.6 × 10^4^ ± 1.5 CFU/mL	
**T1 (15 days)**	1.1 × 10^5^ ± 0.3 CFU/mL	5.8 × 10^4^ ± 2.5 CFU/mL	4.3 × 10^5^ ± 2.3 CFU/mL	2.0 × 10^5^ ± 0.2 CFU/mL
**T2 (30 days)**	1.4 × 10^5^ ± 1.3 CFU/mL	2.9 × 10^4^ ± 1.9 CFU/mL	3.7 × 10^5^ ± 4.4 CFU/mL	3.3 × 10^5^ ± 2.3 CFU/mL
**T3 (90 days)**	4.8 × 10^5^ ± 0.4 CFU/mL	6.2 × 10^5^ ± 0.1 CFU/mL	1.1 × 10^6^ ± 0.8 CFU/mL	2.6 × 10^5^ ± 0.4 CFU/mL

**Fig. 5:**
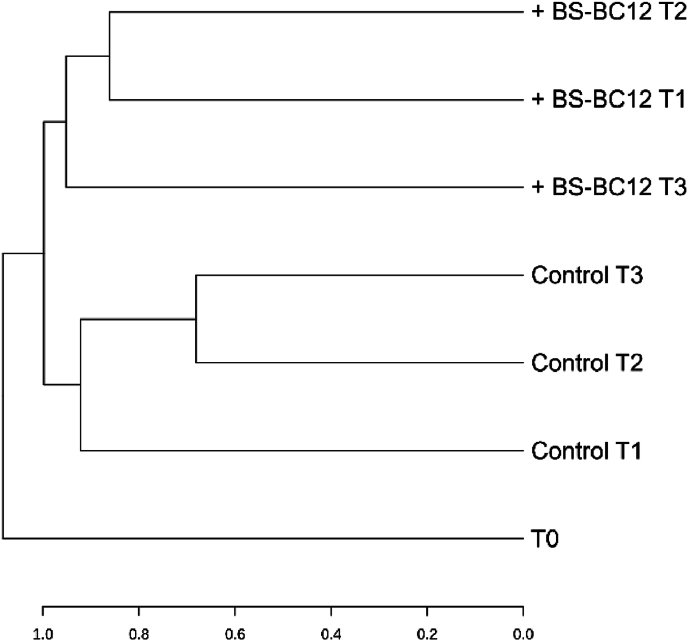
Dendrogram of the microbial communities found in fluvial microcosms based on Bray-Curtis analysis at ASV level.

A phylogenetic NGS approach was used to analyze bacterial community composition of the same microcosm samples. Chao1 and Shannon diversity indices did not reveal any significant effect of BS-BC12 on the richness and diversity of bacterial communities in spring water microcosms at any time point (Table S1). However, the Bray-Curtis analysis indicated that at T1 (15 days), T2 (30 days), and T3 (90 days) time points, the water samples added with BS-BC12 were more similar to each other than to the corresponding control samples. Dendogram in Fig. 5 shows how, at ASV level, the BS-added samples clustered together and separated from the three control samples.

**Fig. 6:**
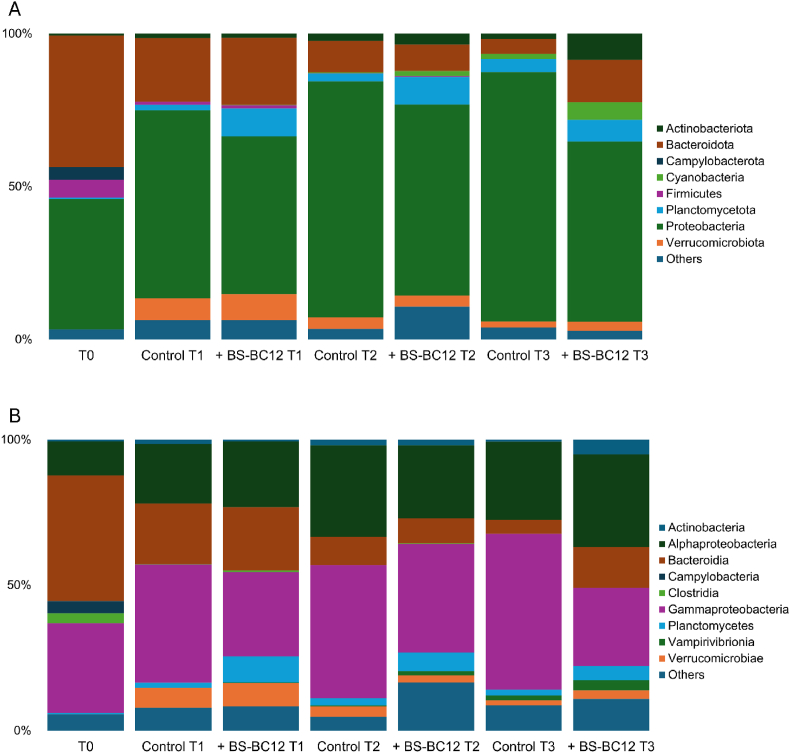
Taxonomy composition (relative abundance) of the microbial communities of fluvial microcosms at phylum **(A)** and class **(B)** levels. “Others” include phyla and classes with abundances <3 % in all samples.

The bacterial flora of the collected freshwater sample (T0) was mainly composed of *Bacteroidota* and *Proteobacteria* phyla (Fig. 6A), both covering 43 % of the total bacterial community. At the class level, *Bacteroidia* dominated (43 % of the total community) followed by *Gammaproteobacteria* (31 %) and *Alphaproteobacteria* (12 %). Additional abundant phyla were *Firmicutes* (6 %) and *Campylobacterota* (4 %), which were mainly composed of *Clostridia* (4 %) and *Campylobacteria* (4 %) classes (Fig. 6B).

After 15 days of incubation in microcosms (T1), slight changes in the relative abundance of some bacterial groups were observed in the control sample. The percentage of the *Bacteroidia* class was halved (21 %), while the percentage of *Gammaproteobacteria* and *Alphaproteobacteria* increased to 41 % and 21 %, respectively. *Clostridia*, and more generally *Firmicutes*, almost disappeared (<1 % of the total community). On the other hand, one additional abundant class was detected, i.e., *Verrucomicrobiae* (7 %). The corresponding (T1) BS-BC12 added sample showed a similar distribution of the bacterial groups. However, some differences were found that included the univariate abundance of *Gammaproteobacteria* compared to T0 sample and the significant increase of the *Plantomycetes* class, which represented 9 % of the total bacterial community.

In 30-day microcosms, both the control and BS-BC12 added samples showed a slight increase of *Gammaproteobacteria* and *Alphaproteobacteria,* together with a decrease of *Bacteoroidia* (<10 % of both total communities) and *Verrucomicrobiae*, this latter representing 4 % in control and 3 % in BS-BC12 added samples. *Plantomycetes* were still abundant in the control microcosm (6 %) and represented only 2 % in the BS-BC12 added one.

Although the *Planctomycetes* and *Alphaproteobacteria* classes were unchanged after 90 days incubation in both control and BS-BC12 samples, *Gammaproteobacteria* increased up to 54 % in control and dropped to 27 % in the BS-BC12 community in T3 microcosms. *Bacteroidia* represented 5 % and 14 % in the control and BS-BC12 samples, respectively, while *Verrucomicrobiae* were found below 3 % in both samples. Two additional abundant classes were detected after 90 days, i.e., *Vampirovibrionia* (belonging to the *Cyanobacteria* phylum), with an abundance of 4 % in the BS-BC12 sample and <2 % in the control sample, and *Actinobacteria*, which represented 5 % of the total community in the BS-BC12 added sample and found in traces (<1 %) in the control microcosm.

Although about 40 % of the total ASVs were not taxonomically classifiable at the genus level, some changes from T0 to the latest timepoint and some differences between control and BS-BC12 added microcosms could be observed. At T0, the most abundant classified genera were affiliated with *Flavobacterium*, *Pseudarcicella* (both of *Bacteroidota* phylum), *Hydrogenophaga*, and *Rhodoferax* (of *Gammaproteobacteria* class) (Fig. S2). These four genera were found only in traces in both microcosms at the latest time point (T3). Further, few bacterial genera were significantly present either in control or in BS-BC12 community at each time point (Table S2). These include *Limnobacter* (*Gammaproteobacteria*) in control samples at T1, *Flavobacterium* (*Bacteroidota*) in BS-BC12 samples at T2, and *Aeromicrobium* (*Actinobacteriota*) in BS-BC12 samples at T3.

## 4 Discussion

In healthy conditions, the vaginal microbiota is primarily dominated by lactobacilli species, such as *L. gasseri*, *L. crispatus*, *L. jensenii,* and *L. iners* [34]. When the vaginal ecosystem balance is disrupted, vaginal infections and dysbiosis can arise. VVC represents a relevant medical issue because of the increasing frequency of *Candida* spp. resistant to azole drugs, which limits the availability of therapies and favours the onset of recurrences [35]. Specifically, RVVC appears to be closely associated with epithelium-based *Candida* biofilms that contribute to the disease persistence and the high resistance to fluconazole [10].

To prevent *Candida* adhesion and biofilm formation, lactobacilli can secrete molecules with antimicrobial properties, including biosurfacatants [36,37]. The anti-biofilm activity toward bacteria and fungi of biosurfactants from vaginal *L. crispatus* and *L. gasseri* strains have been demonstrated [[17], [18], [19], [20]]. In the present work the attention has been placed on *L. gasseri* BC12 since other postbiotics from this strain, namely exopolysaccharides [38] and extracellular vesicles [39], revealed good capability to selectively inhibit biofilm and adhesion of a plethora of pathogens. Here, the biosurfactant from *L. gasseri* BC12 was sought for its anti-*Candida* potential, to broaden the spectrum of biotherapeutics useable for the prevention and treatment of VVC and RVVC.

The chemical characterization of BS from *L. gasseri* BC12, performed through MS, MS/MS and HRMS assays, only identified molecules with relatively low *m/z* values (<1500), corresponding most probably to peptides and peptide fragments; the presence of single amino acid signals confirmed this. The peaks in the FT-IR spectrum are similar to the ones highlighted in other studies where lipopeptidic biosurfactants from lactobacilli non-*gasseri* were investigated [20,40,41]. Although no exhaustive data on the BS structure was obtained, some key information has been retrieved. Detailed chemical characterization of BS from lactobacilli is well-known as an intricate task, and just a few isolated examples of successful characterization can be found in the literature [42]. For example urfactin, iturin and lichenysin were identified in vaginal *Lactobacillus* spp. by high-resolution liquid chromatography electrospray ionization mass spectrometry [37]; *L. acidophilus* BS was identified as glycolipoprotein type by thin layer chromatography and Fourier transform infrared spectroscopy [43]. In general, it is necessary to implement specific knowledge on the chemistry of lactobacilli BS and it will be our interest and priority to develop tools and methods to achieve this aim [[44], [45], [46]].

*L. gasseri* BC12 biosurfactant proved to have tensioactive properties with a critical micellar concentration value of 1.2 mg/mL. This property, along with the emulsification properties observed for 3 oils (olive oil, sunflower oil, and wheat germ oil), are in line with what was previously reported for other lactobacilli’s biosurfactants [19,20,47,48].

*Candida* species have numerous virulence factors, such as the ability to adhere to the host epithelium strictly and to form biofilm, which are crucial to promoting the spread of infection. The biofilm formation is directly connected with the outbreak of VVC and especially of RVVC, making *C. albicans* adapt to adhere to abiotic surfaces, like many medical implants [2,49]. Clinical treatments of fungi infection are really challenging due to the biofilm formation itself, representing one of the main virulence factors that contribute to candidiasis pathogenesis [50], antifungal drug resistance [51,52] and recurrence [10].

Considering the crucial role of biofilm in candidiasis, the capability of BS-BC12 to impair *Candida albicans* and non-*albicans* biofilm formation and to disperse mature biofilms was investigated. Since the biological activity of a tensioactive may depend on its organization as micelle in solution, BS-BC12 was tested at CMC value and its half.

The anti-biofilm properties of lactobacilli BS toward different opportunistic pathogens (like *E. coli*, *S. aureus*, etc.) and sexually transmitted pathogens (like *N. gonorrhoeae*) were investigated in many studies [34,36,48]; other studies report the anti-biofilm activity specifically against *Candida* [18,37,53]. In this scenario, the inhibitory activity of crude BS-BC12 was more robust and more extensive in terms of the spectrum of action compared to the literature data. We noticed that BS-BC12 at 1.2 mg/mL was more active toward *C. albicans* biofilm (inhibition rates: 88 %–93 %) while the concentration of 0.6 mg/mL was more active on non-*albicans* biofilm (inhibition rates: 42–50 %). Because of the high frequency of *C. albicans* in VVC/RVVC cases and how challenging it is to cure candidiasis infections [3,54], these high inhibition rates become very promising. Non-*albicans* species, nowadays, are increasingly represented in candidiasis cases, making these infections even more challenging to be defeated [35,55]. For this reason, the anti-biofilm activity exploited by BS-BC12 (0.6 mg/mL) toward *C. glabrata* strains and *C. lusitaniae* is also noticeable, suggesting that this BS has a wide-spectrum of anti-*Candida* activity.

In the present study, we also tested the ability of BS-BC12 to disperse pre-formed *Candida* biofilms. The eradication process of the biofilm is known to be much more challenging than the inhibition one [56]. *Candida* biofilms are highly resistant to the majority of known antifungal drugs due to a multifactorial phenomenon. Among these factors, the presence of an extracellular matrix plays a key role in providing physical protection from antifungal compounds and drugs and helping yeast evasion from host immunity [56,57]. Our results confirmed that BS-BC12 at the CMC concentration (1.2 mg/mL) is the most effective toward *C. albicans* strains (eradication rates: 30–46 %), while BS-BC12 at half CMC (0.6 mg/mL) is more effective on *C. glabrata* and *C. lusitaniae* strains (35-25 %). Overall, data from biofilm formation and eradication assays suggest that the anti-biofilm activity of BS from *L. gasseri* BC12 is dose- and species-dependent. We can speculate that this dependence is linked to the self-aggregation ability of BS determined by CMC. At a concentration of 1.2 mg/mL the BS form micelles, while at a concentration of 0.6 mg/mL the BS is free in solution. It appears that BS-BC12 is more active on *C. albicans* strains in micellar form, while its free form is more effective on non-*albicans* strains. This diversified anti-biofilm profile of BS-BC12 may be due to the specific surface characteristics of biofilms formed by the different *Candida* species, whereby the interaction of the BS with these structures may depend on the aggregation state of the BS itself (micelles or not). However, considering that no fungicidal effects were observed, the anti-biofilm activity of BS-BC12 is likely due to the capacity of biosurfactant to reduce the hydrophobicity of abiotic surfaces, which consequently are less susceptible to pathogens’ adhesion.

Notably, in the dispersal assay, BS-BC12 was less effective than in the inhibition one, probably due to the biofilm characteristics themselves and the difficulty of this molecule to penetrate the extracellular matrix, in agreement with what was already noticed in a previous study on a different biosurfactant [18]. However, BS-BC12 induced significant biofilm dispersal on 8 out of 10 *Candida* strains, suggesting that BS-BC12 could be employed for the prevention but also for the treatment of an ongoing yeast infection. Considering that, when proposing a new therapeutic agent, the safety profile is a priority. Thus, the effects of BS-BC12 on human epithelial cells were evaluated in order to exclude any cytotoxicity. Overall, BS from *L. gasseri* BC12 was proven safe on HeLa cells, although after 48 h of incubation, it determined a significant decrease in cell viability at all concentrations tested (9–20 %), likely due to a detergent-like effect which can lead to breaks in cell membrane [47]. However, according to ISO 10993–5:2009, which is applied when evaluating medical devices safety, if the effect toward cells viability is less than 30 %, the compound is considered not cytotoxic.

Upstream the biofilm formation, *Candida* spp. can adhere strictly to host epithelial cells. The adhesiveness is due to adhesins, which are surface proteins that allow yeast cells to adhere to all types of surfaces, both biotic and abiotic [58,59]. Biosurfactants are amphiphilic and surface-active compounds that affect surface tension. It is widely known that BS have a vigorous anti-adhesion activity toward many pathogens, including *Candida* [36]. De Gregorio et al. [20] focused on BS from vaginal *L. crispatus* BC1, demonstrating its antagonistic effect toward the adhesion of clinically isolated *Candida albicans* and non-*albicans* species (*C. albicans, C. glabrata, C. krusei* and *C. tropicalis*) from VVC infected women on cervix epithelial cells. The exclusion method resulted in the most effective one toward *Candida* spp [20].

The results from the present study showed that BS from the vaginal strain *L. gasseri* BC12 was able to reduce the adhesion of all *Candida* strains tested in the exclusion assay and of 9 *Candida* strains out of 10 in the competition assay. This suggests that BS-BC12 effectively prevents and treats *Candida* adhesion. Notably, in the anti-biofilm experiments, no significant effect was registered on *C. krusei* and *C. parapsilosis*, while they were sensitive to BS-BC12 treatment in both the adhesion assays. The reason behind the anti-adhesive effect of BS-BC12 is probably linked to the chemical nature of this compound that may alter the hydrophobicity of cells, reducing the adhesion of many microbial organisms [60].

Few studies assessed the impact of surfactants on bacterial communities in environmental samples, particularly in freshwater (rivers, lakes). A recent study conducted to evaluate the effects of biological and synthetic surfactants on a freshwater biofilm community indicated that both surfactants had adverse effects on the communities in terms of reduction of microbial diversity, with the latter also reducing the abundance of many taxa [61]. Conversely, we demonstrated that BS-BC12 provided no significant reduction of the bacterial richness (Chao1) and diversity (Shannon); rather, it seemed to provide a slight increase on both indexes. Additionally, no evident alteration of the taxonomic composition was observed at all time points, as only a few bacterial genera were significant enriched either in control (*Limnobacter* at T1) or in BS-BC12 (*Flavobacterium* at T2) community. *Limnobacter* commonly populates lakes/rivers sediments and freshwaters and is known to be involved in denitrification processes [62]. *Aeromicrobium* and *Flavobacterium* are both chemoorganotrophs and inhabit freshwater environments, the latter being capable of dissolving and mineralizing high molecular weight organic matter [63]. These minor changes may be related to the biodegradation of BS as a source of C/N, which may have influenced the relative abundance of these few bacterial genera in the different microcosms. Some studies report using different biosurfactants as nutritional sources by microorganisms [64,65]. The potential environmental biodegradability of BS from *L. gasseri* BC12 offers an additional element to its biocompatibility and possible further applications.

## 5 Conclusions

This study demonstrates the anti-*Candida* activity of the biosurfactant recovered from vaginal *L. gasseri* BC12. BS-BC12 was characterized chemically and revealed a typical lipopeptide structure with a predominance of peptide portion. BS-BC12 showed good emulsification properties against different substrates with a low critical micellar concentration (1.2 mg/mL), in line with its chemical features. This biosurfactant, according to ISO 10993–5:2009 valid for medical devices, was considered not cytotoxic for HeLa cells. The anti-*Candida* activity of BS-BC12 was demonstrated against both *C. albicans* and non-*albicans* strains in terms of anti-biofilm effects and capacity to reduce yeast adhesion to HeLa cells. The environmental impact of BS-BC12 on spring water microcosm was evaluated and no significant differences were noted in the microcosms with BS-BC12 compared to the control samples, in terms of total culturable bacterial counts. In addition, no remarkable modifications in the taxonomy composition of the bacterial ecosystem were also seen by the NGS 16S rRNA gene analysis. To conclude, this study represents the first chemical and functional characterization of BS recovered from *L. gasseri* BC12. For the first time, environmental impact of lactobacilli BS was brought about. However, further studies are necessary to investigate deeper the BS-BC12 potential use in humans.

## CRediT authorship contribution statement

**Federica Monti:** Investigation, Data curation, Methodology, Writing – original draft. **Barbara Giordani:** Investigation, Writing – review & editing, Formal analysis, Methodology. **Stefano Fedi:** Methodology, Resources, Writing – review & editing, Investigation, Formal analysis. **Daniele Ghezzi:** Writing – original draft, Investigation, Data curation. **Paola Galletti:** Investigation, Methodology, Resources, Formal analysis. **Laura Mercolini:** Resources, Investigation, Writing – review & editing, Methodology. **Roberto Mandrioli:** Methodology, Writing – review & editing, Resources, Investigation. **Carola Parolin:** Investigation, Formal analysis, Writing – review & editing. **Barbara Luppi:** Writing – review & editing, Supervision, Resources, Conceptualization. **Beatrice Vitali:** Project administration, Supervision, Writing – review & editing, Resources, Conceptualization.

## Data availability

Data of technological characterization of BS from *L. gasseri* BC12 (surface tension and emulsification properties), anti-*Candida* assays (anti-biofilm and anti-adhesive assays) and MTT assay are published in the AMS acta repository (https://doi.org/10.6092/unibo/amsacta/7683). 16S rRNA sequences generated in this work are published in the SRA database under accession number PRJNA1109740.

## Declaration of competing interest

The authors declare the following financial interests/personal relationships which may be considered as potential competing interests: Beatrice Vitali reports financial support was provided by 10.13039/501100004740Fondazione del Monte di Bologna e Ravenna. If there are other authors, they declare that they have no known competing financial interests or personal relationships that could have appeared to influence the work reported in this paper.

## Data Availability

I have reported the codes of my data in the manuscript
