# Supplementary material for: Vaginal Lactobacillus gasseri biosurfactant: a novel bio- and eco-compatible anti-Candida agent
Source: Biofilm. 2025 Jun 24;10:100299. doi: 10.1016/j.bioflm.2025.100299 (PMC12269986; doi:10.1016/j.bioflm.2025.100299)
Supplement: Multimedia component 1 [file mmc1.docx]

**SUPPLEMENTARY MATERIALS**

**Fig. S1** FT-IR spectrum of biosurfactant isolated from *L. gasseri* BC12.


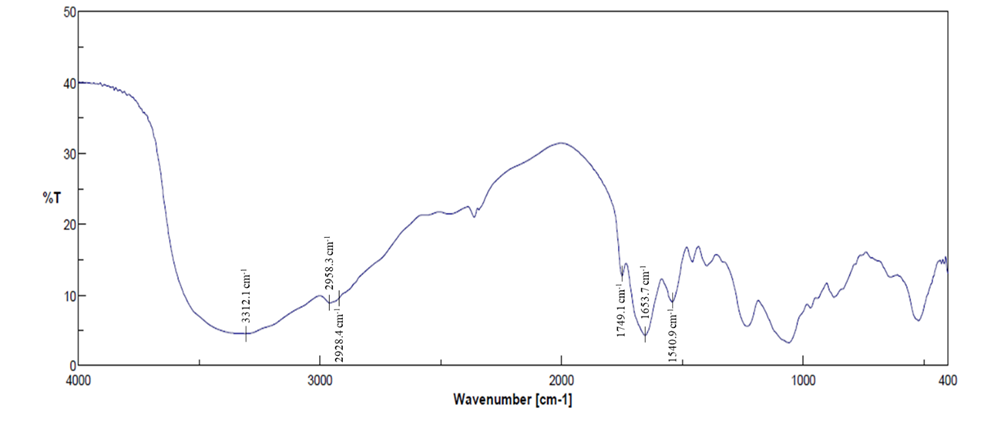


**Fig. S2** Heatmap showing the distribution of the most abundant taxa (> 2% in at least one sample) among the microbial communities of fluvial microcosms.


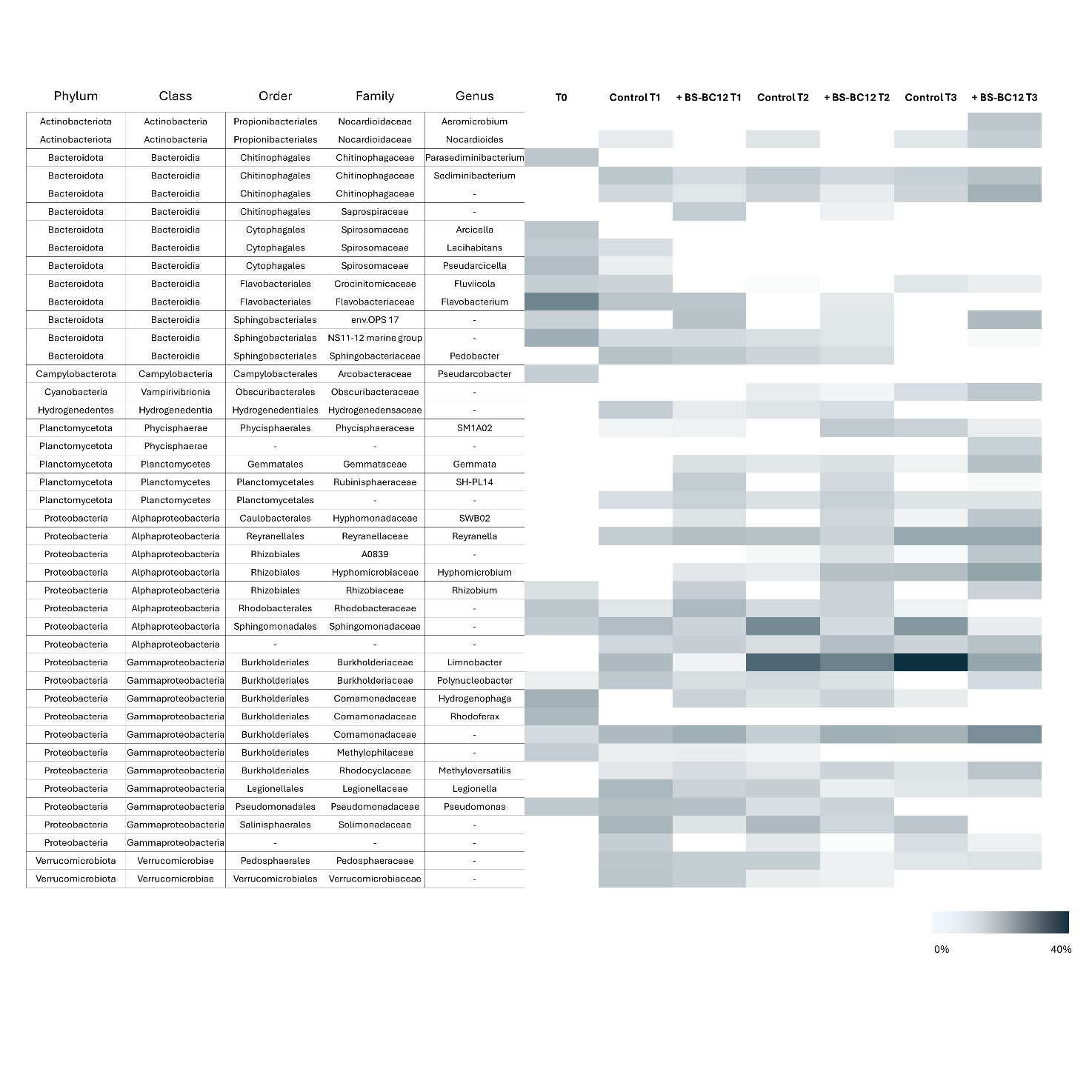


**Table S1** Alpha diversity indexes of the microbial communities in the fluvial microcosms under analysis.

| **Timepoint** | **Microcosm** | **Chao1** | | **Shannon** | |
| --- | --- | --- | --- | --- | --- |
|  |  | ***index*** | ***p-value*** | ***index*** | ***p-value*** |
| T0 | T0 | 122 | - | 4.60 | - |
| T1 | Control T1 | 228 | ≥ 0.05 | 5.03 | ≥ 0.05 |
|  | + BS-BC12 T1 | 268 |  | 5.29 |  |
| T2 | Control T2 | 237 | ≥ 0.05 | 4.97 | ≥ 0.05 |
|  | + BS-BC12 T2 | 340 |  | 5.45 |  |
| T3 | Control T3 | 221 | ≥ 0.05 | 4.27 | ≥ 0.05 |
|  | + BS-BC12 T3 | 259 |  | 4.39 |  |

**Table S2** Bacterial genera significantly enriched either in BS-BC12 or in control microcosms at different timepoints.

| **Timepoint** | **Microcosm** | **Genus** | **p-value** |
| --- | --- | --- | --- |
| T1 | Control T1 | Limnobacter | 0.018 |
| T2 | + BS-BC12 T2 | Flavobacterium | 0.010 |
| T3 | + BS-BC12 T2 | Aeromicrobium | 0.049 |
